# Address of the Retiring President A. D. M. S., Thomas Wooten, M. D., Austin

**Published:** 1888-10

**Authors:** 


### PRESIDENT WOOTEN'S ADDRESS.

**Delivered Before the Austin District Medical Society at its Annual Meeting, September 20, 1888.**

Voted to be Published in Daniel's Texas Medical Journal.

*Mr. President, and Gentlemen of the Austin District Medical Society:*

Prompted by a spirit of progress, a few devotees of medicine conceived the idea of organizing a District Medical Society in the interest of medical science and professional progress. A little over one year ago this idea culminated in a circular letter of invitation to physicians, embraced in five counties convenient to the city of Austin, to participate in the formation of such a society, and fixed the time of meeting September 8, 1887, and the city of Austin the place. In response to this call, twenty-four physicians assembled; our worthy confrere, Dr. W. A. Morris, was invited to the chair, and called the meeting to order. After preliminary matters, discussion of constitution and by-laws, a society was duly organized, (and christened, "Austin District Medical Society,") by the election of officers for the ensuing year. The election resulted as follows: Thos. D. Wooten, president; W. T. Richmond, first vice-president; L. H. Hardy, second vice-president; T. J. Bennett, secretary. Such, gentlemen, is a brief synopsis of our Society.

By virtue of the highly appreciated honor conferred upon me on that occasion, I appear before you to-night to deliver an address required of your retiring President. In reviewing the year of the infantile history of the Austin District Medical Society, I think there are numerous and valid reasons why it should receive from us hearty congratulation. We have witnessed its growth, progress and success. The Society numbers fifty members at the present time. If we will review its year's work, and reread the articles emanating from its members during the short period of its existence, and then could have before us, complete, in print, the discussions elicited by its papers, we would have quite a medical volume of scientific and practical value, worthy of earnest perusal and after-consideration. In addition to this, if we could accurately estimate the reading, research and thought prompted and inspired by its papers and discussions, we might then have only an approximate appreciation of the great worth and value of the year's work, and the great value and importance of medical societies in general; and might furnish a slight forecast of what may come out of the Austin District Medical Society, to the honor and glory of medicine, in the years that roll on. I trust that every member present, new and old, will renew his allegiance to the Austin District Medical Society, to-night, in a most earnest manner, it being the ending of the old, and the beginning of a new year, that we may be united in friendship and bound together in one common social and professional brotherhood.

Our society is an integral part of the medical profession in Texas; and the entire profession owe important public obligations to society which should secure earnest consideration by every patriotic doctor in the State. In this connection I would suggest that it is a wise custom among successful business men to take an inventory of stock, and make a retrospect of the run of business. I think the example a good one for the doctors, and it would prove well for the doctors of Texas to come to a halt, make a professional retrospect and take their reckoning, examine chart and compass, and determine their bearings and relations to society and the State.

There is probably no observing doctor who has lived long enough in Texas to embrace that dark, historic period that followed after the war, who remembers the chaotic, unorganized condition of the medical profession of that period, whose heart does not swell with pride and gratitude at the present outlook and aspect of medicine in Texas, compared to what it was in those years long gone by. The medical profession of Texas has been, and is, progressive; not only has it increased in numerical strength, but has grown in scientific and practical professional knowledge; has increased and is developing in scientific thought, investigation and research.

About twenty years ago, the State Medical Association was organized, and has accomplished an efficient and noble professional work. County and district medical societies have been organized all over the State; a healthful rivalry has been inspired in scientific investigation and research, evinced by the many papers and publications that have come to light in the records of the State Association, and the Society publications that have appeared in the medical journals. We have three medical publications in the State, two of which have existed for several years. The third one is of more recent date. They lack the prestige of age, the contributions and the experience of journalism. They are, however, a beginning, and are a credit to the profession and our State. In a review of these productions that have emanated from these several sources, we find, with a little culling, that they constitute and form a very respectable beginning in the way of medical literature, of which posterity may not be ashamed.

The medical profession of Texas in part have done well. They have as a class been in earnest, and faithful in personal attention to their patients, and in the main they have shown themselves self-sacrificing and have exercised that kind, humane philanthrophy that has ever characterised the true physician. I do not believe they stand blamable in this regard. Possibly they have been too exclusively occupied by these personal, local professional obligations, and have failed in giving attention to the weightier matters of concern due society and the general public welfare. Does not a candid review of the past and consideration for the present prove that the doctors of Texas have been wanting in true professional patriotism, in that they have left unaccomplished matters of great concern to the health and well-being of the commonweath, that a patriotic, united influence upon their part would have carried into effect? The practicing physicians in Texas number about four thousand. Those, whose names appear in the organized work of the profession, number between four and five hundred. Only about eight or ten per cent. have been engaged in the concerted, organized professional work. The problem suggests itself, if eight or ten per cent. have performed what has been accomplished, what would have been the result, if the ninety or ninety-two per cent. had been likewise engaged in this organized professional work. The State's record clearly indicates that the public welfare sadly needed the earnest patriotic influence of every doctor in the State. This leads up to the question that deserves solution. What is our professional standing before the body politic of Texas? Have we any standing? Is there a single law or statutory provision in the statutes of Texas that emanates from or that is a reflex of the genius and intelligence of the medical profession of Texas?

This is a broad proposition, and it may, on the moment, be questioned, from the remembrance of some of you, that the State had a law to regulate the practice of medicine. So it did. I acted as chairman of the committee appointed by the State Medical Association, and drafted the bill. The law, as it was originally enacted, was a credit to the profession and the people of the State. It placed Texas in advance of every State in the Union, in the protection of her people against the quackery and charlatanism of the age. It was so recognized and favorably commented upon by the intelligent people and the profession throughout this country. Compare the present law, as it now stands, with the original law; you find it completely emasculated, and worse than no law at all, because it fastens upon the people cercificate doctors, given often by incompetent boards to men without professional education or training. Our State may claim an efficient quarantine law. It may be so, but it is largely a political machine, and has never been submitted to the surveillance and that sagacity that is supposed to be derived best from medical minds. Has the State a board of health, or a medical commission, or a committee of intelligent physicians? a requirement thought to be necessary in thirty-three States of this Union, for the better protection of their people, by prevention of disease in having regard to sanitary law. Who is it that examines the sanitary conditions of our asylums, and pronounces upon the competency of their management? Who visits the penitentiary, prisons, jails, hospitals, and looks after the healthful condition and construction of school houses and other public buildings, and acts as an advisory council in other matters relating to public health ? The medical profession of Texas, as far as represented, have not been remiss in their fealty to the public in these matters. The State Medical Association have had a standing committee on legislation, and have again and again, for years, presented these matters for legislative action, without a practical hearing before the legislature. A special committee was appointed by the Association at Galveston, to wait upon the Twentieth legislature. After the completion of the State capitol, a building of which every true Texan feels a just pride, a house truly of many mansions, in which every department of State was expected to find an abiding place, a room, or rooms, was politely asked to be set apart for the Health and Medical Department of the State, a depository for a State Medical Library, and a place for State medical literature, a museum, etc. This polite and reasonable request was met by a motion of ridicule that a like apartment be given for the boot blacks. This insult to the physicians of the State was passed by without rebuke. It may be of satisfaction to know that the author has been relegated to political oblivion.

I believe it is an accepted, though a melancholy fact, that the medical profession has not the hold upon public confidence and favor, in Texas or elsewhere in America, it had years ago. The causes and influences that have contributed to this result are worthy of much thought, and should elicit the earnest consideration of every honest and true devotee of rational medicine, that the confidential relationship may be reinstated and the profession occupy its true position of trust, honor and dignity, commensurate with the sacred duties and responsibilities the calling imposes. This want of confidential regard for the medical profession by the public is manifested often on public occasions. It seems to have been shown especially upon the very noted meeting, at Washington City, of the International Medical Congress, a congress composed of the greatest medical minds of the world. These medical savants were specially concerned in the discussion of topics in the domain of State or preventive medicine, matters of vital importance to all mankind, and one supposed to be of interest to all educated people. The disinterested benevolence of the medical profession was peculiarly manifested in this congress. It embraced doctors from all the civilized countries of the globe, putting distance behind them, sacrificing pleasure, interest, time and money, to meet in the interest of humanity; yet, the occasion was obviously unappreciated by the American people. Prof. Roberts Bartholow makes this the subject of his able address in medicine, before the American Medical Association at Cincinnati, in which he narrates some of the causes which have contributed to this result, an extract from which I take the liberty of making: "Not a little of the ignorance, the crudities, the vague theories of the eighteenth century still linger in the nineteenth century near its close. It is yet understood by many that the therapeutic art is based on some ism or dogma. 'Allopath' and 'homeopath' is still bandied about as having vital power, when they were long since relegated to the lumber-room of the past by modern science. We yet hear of 'new school' and 'old school,' as if notions brought out near the close of the last century, compounded of the vagaries of Hahnemann, and the mysticism of Messmer, could still continue guides to practice. The contentions of so-called 'schools,' the noisy demonstration of the Thompsonians, and eclectics, and the physio-medicals; the rapid water-cure, and the innumerable special cures, have produced in the popular mind, a distrust of all systems."

"The success of such wretched puerilities, such inanities as the homeopathic practice consists of, does more to lower the position of the medical profession than any other cause."

I fully endorse the truth of Dr. Bartholow's views and could add to the list of "wretched puerilities and inanities" by him enumerated, which come in likewise for a full share of prejudicial influence upon the public mind. It will apply, indeed, to all frauds and impostors upon public confidence, made by medicine, or in the name of medicine; by it the fraudulent vendor or patentee quack-doctor or lay member it passes to the discredit of the medical profession, by the deceived and defrauded people. Patent medicine vending is a moral blight upon the truth and honor of medicine, and their indisrciminate use by the public is in violation of the fundamental principles of therapeutics, in that medicinal agents are relative in their action upon disease, and not specifics to prove curative; thererore they must be given or used with a special regard to their physiological action upon a pathological condition.

There is an inherent, or an acquired propensity in the nature of a large portion of mankind, to take medicine often for fancied diseases, that do not exist. When this class is appealed to, this propensity stimulated into exercise by the ingenious advertisements of the unscrupulous, mercenary quack nostrum vendor, their credulity becomes morbid beyond all reason. They are not content in taking their favorite panaceas and nostrums, but they become infatuated, and they seek out their credulous neighbors and friends to participate in the blessings of their infatuation and folly. This wave of human credulity and infatuation has struck this community badly, and its phenomenal mode of action is beautifully illustrated in the history, as well as nature of the "Microbe Killer," an infernal sulphurous decoction, compounded to kill and exterminate worms, insects, and all other vermin, enemies to the health and vigor of the vegetable kingdom. In this mortal field of combat, it proved a swift agent of destruction, a credit to its compounder and happy hit for the vegetable kingdom. Like Perkinson, in the earlier days of electrical discovery, the "Microbe Killer" struck a tidal wave; it has made its debut in the period of microscopic discovery, when every disease is supposed to have micro organism, bacteria or cocti; so much having been said and written of a mysterious and occult nature in regard to microscopic organisms, as to constitute this the microbean age, it was very natural for somebody to find an universal and an infallible microbe killer for the whole tribe. Medical men are expected to be the custodians of the public health, and we are the advocates of preventive medicine; as such can we be true to ourselves, to our profession and to our people and sit serenely by and see our people swindled and injured in health by gulping down this worthless compound? We know its origin, we know its composition, and know it to be a therapeutic heresy. Do not honor and justice demand our protest? and the people are entitled to our warning. What must be the feelings of every honest Texas doctor in visiting his Capital city and passing up the its streets, to find its wall emblazoned and its heights painted in monumental form—charlatanism and quackery rampant. If he has imbibed the least bit of the spirit of the Hippocratic oath, he will realize a loathing at the pit of his professional stomach, and hang his head in shame.

If the medical men of the State of Texas expect ever to acquire the influence needful to secure the legislation for their State demanded by the enlightened civilization of the times, they must organize, co-operate, and agree upon a simple, well-defined policy as to what legislation is required for the good of the people of this commonwealth, in matters relating to public health, and their protection against the charlatanism and quackery of this age. While the profession should act together as a unit in a common cause, the success depends upon the personal and individual influence exerted at home among his patrons and friends, by each and every member of the profession. Teach them what is needed for their welfare and protection.

If we regain the professional favor and confidence of the people, we must do so by the individual exercise of those exalted principles of head and heart that make us honest, kind, humane and philanthropic. The personality of the doctor, his disposition, his habits and his character enter largely into his success and popularity. We should ever be in earnest, and give tireless attention to professional work and duty. We should study that we may progress and advance, by revising old methods, testing the new, and holding fast to the best.

